# Nanoparticle Delivery of Natural Products in the Prevention and Treatment of Cancers: Current Status and Future Prospects

**DOI:** 10.3390/cancers3044024

**Published:** 2011-10-26

**Authors:** Dhruba J. Bharali, Imtiaz A. Siddiqui, Vaqar M. Adhami, Jean Christopher Chamcheu, Abdullah M. Aldahmash, Hasan Mukhtar, Shaker A. Mousa

**Affiliations:** 1 The Pharmaceutical Research Institute at Albany College of Pharmacy and Health Sciences, 1 Discovery Drive, Rensselaer, NY 12144, USA; E-Mail: dhruba.bharali@acphs.edu; 2 Department of Dermatology, University of Wisconsin, Madison, WI 53706, USA; E-Mails: isiddiqui@dermatology.wisc.edu (I.A.S.); vmadhami@wisc.edu (V.M.A.); jcchamcheu@dermatology.wisc.edu (J.C.C.); hmukhtar@wisc.edu (H.M.); 3 Stem Cell Unit, College of Medicine, King Saud University, Riyadh, 11461, Saudi Arabia; E-Mail: dahmash@ksu.edu.sa (A.M.A.); 4 University Hospital of Odense & Medical Biotechnology Center, Winslowsparken 25, DK-5000, Odense, Denmark

**Keywords:** nanoparticles, cancer, chemoprevention, natural product, epigallocatechin-3-gallate, curcumin, resveratrol, taxol, camptothecin

## Abstract

The advent of nanotechnology has had a revolutionary impact on many aspects of 21^st^ century life. Nanotechnology has provided an opportunity to explore new avenues that conventional technologies have been unable to make an impact on for diagnosis, prevention, and therapy of different diseases, and of cancer in particular. Entities in nanometer sizes are excellent platforms to incorporate various drugs or active materials that can be delivered effectively to the desired action site without compromising the activity of the incorporated drug or material. In particular, nanotechnology entities can be used to deliver conventional natural products that have poor solubility or a short half life. Conventional natural products used with entities in nanometer sizes enable us to solve many of the inherent problems (stability, solubility, toxicity) associated with natural products, and also provide a platform for targeted delivery to tumor sites. We recently introduced the novel concept of using nanotechnology for enhancing the outcome of chemoprevention, which we called ‘nanochemoprevention’. This idea was subsequently exploited by several laboratories worldwide and has now become an advancing field in chemoprevention research. This review examines some of the applications of nanotechnology for cancer prevention and therapy using natural products.

## 1. Introduction

Nanotechnology is an emerging, multidisciplinary field that frequently employs techniques and tools from diverse disciplines, including biology, engineering, chemistry, and medicine. Nanotechnology is typically known as the study of the control of matter on an atomic and molecular scale, generally structures in the nanometer (10^−9^ m) range, and involves developing materials or devices on that scale. The basic idea behind nanotechnology is that metal, semiconductor, and polymeric nanoparticles have novel optical, electronic, magnetic, and structural properties that are often not available from individual molecules and bulk solids [1,2]. In recent years, nanotechnology has been assessed and implemented in different areas of cancer management and therapeutics with the hope that it will lead to major advances in cancer diagnosis and treatment [1,3-6].

Most biological processes, including those that are cancer-related, occur on a nanometer scale, and thus nanoparticle technology has been greatly appreciated as a potential tool for cancer diagnosis and treatment, a field of science generally referred to as ‘cancer nanotechnology’. Scientists in this field seek to describe the relation of nanoscale materials and devices to cellular and molecular components specifically related to cancer. Cancer nanotechnology offers nanoparticles designed to target tumors (passively and/or actively) and increase the solubility and bioavailability of attached drugs in order to administer novel therapies [7]. Nanoscale materials and devices with unique therapeutic properties can be engineered to deeply infiltrate tumors with a high level of specificity. Cancer nanotechnology is acknowledged by the National Cancer Institute, which considers that nanotechnology offers an extraordinary, paradigm-shifting opportunity to make significant advances in cancer diagnosis and treatment [5].

Since prehistoric times, humans have used natural products (natural active ingredients) to treat various diseases. In modern times, natural products have been used for the prevention and treatment of cancer, still an elusive goal in medicine. Numerous natural products have been studied to determine the molecular pathways for cancer prevention and treatment [8-11], including β-carotene, curcumin, epigallocatechin gallate, genistein, resveratrol, gingerol, and capsaicin. Though it has been proven that many natural products have a strong therapeutic value, their poor solubility and bioavailability have severely limited their use. In last few decades scientists have used nanoparticle-delivered, naturally synthesized entities for the treatment and prevention of various cancers.

Over the last few years our laboratory has been actively involved in synthesizing biodegradable nanoparticles encapsulating various natural products including resveratrol, epigallocatechin-3-gallate (EGCG), green tea extract (GTE), and pomegranate extract. We were the first group to introduce the novel concept of using nanotechnology to improve the outcome of chemopreventive intervention and coined the term ‘nanochemoprevention’ [12], which was used to refer to blending ultramodern nanotechnology and natural products like EGCG. Since then, several laboratories worldwide have taken up this concept and many natural products have been used for nanochemoprevention [12-14]. Despite outstanding advancements in fundamental cancer biology and chemoprevention using natural products in preclinical settings, they have not translated into progress from ‘bench to bedside’ for human use. Some of the reasons behind the lack of success of chemoprevention using natural products in clinical trials are: (i) diverse genetic backgrounds of patients at risk; (ii) varied food habits among patients, and more importantly; (iii) inefficient systemic delivery and poor bioavailability of active agents. In order to achieve a maximum response of natural products as chemopreventive agents for human use, strategies are required that can bypass these limitations. Strategies that could lead to sustained release of the chemopreventive agents could critically improve their bioavailability, and in turn reduce the toxicity associated with the high doses that are typically required for optimum response. In this article we present the available data for some of the extensively studied natural products using nanotechnology: EGCG, curcumin, resveratrol, taxol, and camptothecin.

## 2. Epigallocatechin-3-gallate (EGCG)

In our proof-of-principle study of exploiting nanotechnology to increase the systemic delivery and bioavailability of any natural product, we employed nanoparticle-mediated delivery for sustained release of epigallocatechin-3-gallate (EGCG), a polyphenol from green tea [12]. We encapsulated EGCG in poly(L-lactide)-poly(ethylene glycol), (PLA-PEG), nanoparticles and assessed its efficacy against human prostate cancer (PCa) cells both *in vitro* and *in vivo*. The results showed that encapsulated EGCG retains its biological effectiveness with an over 10-fold dose advantage for exerting its efficacy. Advantages of an ecofriendly polymer like PLA-PEG are its history of safe use, proven biocompatibility, and ability to control the time and rate of polymer degradation, and thus it potentially has more clinical relevance. The presence of a hydrophilic polymer like PEG increases the circulation time of the nanoparticles by sterically stabilizing them against opsonization [15]. It was also observed that the nanoparticles can release EGCG in a sustained manner. Thus, sustained release can lead to a lower dose requirement (as observed in our experiments, we used a 10 times lower dose of EGCG in nanoformulation) and could be a valuable tool to limit the toxicity associated with a high dose of active materials/natural products. At the same time, sustained release is likely to enhance EGCG's bioavailability, and thus can reduce the chance of repeated use, which is a must for all anticancer drugs in cancer therapy.

Shutava *et al.* [16] reported a protein/polyphenol microcapsules of EGCG and type A gelatin using the layer-by-layer (LbL) assembly method. Nanoparticle-encapsulated EGCG retained its biological activity and blocked hepatocyte growth factor (HGF)-induced intracellular signaling in the breast cancer cell line MBA-MD-231 as potently as free EGCG [16]. EGCG in the LbL assembly was shown to retain its antioxidant activity, and the kinetics of the reaction of 2,2′-azinobis(3-ethylbenzothiazoline-6-sulfonic acid) diammonium salt (ABTS) cation-radicals with films consisting of 1:10 gelatin/EGCG bilayers were observed to be affected by film structure. The EGCG content in the protein/polyphenol film material was as high as 30% w/w [17]. Polyphenols like EGCG, tannic acid, curcumin, and theaflavin were encased into gelatin-based nanoparticles consisting of a soft, gel-like interior with or without a surrounding LbL shell of polyelectrolytes assembled using the LbL method.

A recent study was done with the purpose of designing and characterizing two flavonoid-loaded lipid nanocapsules (LNC) by applying the phase inversion process to enhance their apparent solubility and/or stability [18]. It was observed that quercetin-loaded LNC30 (3%) and LNC60 (2%) carried a particle size of 30.3 and 55.1 nm, respectively, and had significantly higher entrapment efficiency. Encapsulation of quercetin in LNC enabled its apparent aqueous solubility to increase by a factor of 100 when compared to the free quercetin. In addition, colloidal suspensions proved to be stable in terms of encapsulation for at least 10 weeks, and quercetin was not oxidized. With simple chemical modification of (-)-EGCG, it was possible to reach very high encapsulation rates (95%). A stable colloidal suspension of (-)-EGCG in water was obtained over 4 weeks, while free (-)-EGCG solubilized in water exhibited 100% degradation within 4 hours.

The preparation, activity, and *in vitro* targeting ability of EGCG incorporated in bovine serum albumin (BSA) nanoparticles (NP) has been evaluated in PC-3, a human prostate cancer cell line. The folate-mediated EGCG-BSA nanoparticles' (FA-EGCG-BSANP) morphology and particle size distribution were uniform and even, with a mean particle size of 200 nm. The FA-EGCG-BSANP uptake by cultured PC-3 cells was 23.65 times the amount of EGCG-BSANP in a concentration-dependent manner. The lethality of PC-3 cells treated with FA-EGCG-BSA was 82.8%, with EGCG was 58.6%, and with EGCG-BSANP was 55.1%. Lethality of PC-3 cells was positively correlated with the nanoparticles' uptake amount [19].

It has been suggested that encapsulation of various green tea catechins in chitosan nanoparticles enhances their intestinal absorption and that encapsulation may be a promising strategy for improving their bioavailability [20]. In a recent study poly(lactide-co-epsilon-caprolactone), (PLCL), was successfully developed as an EGCG-eluting polymeric stent, which could be utilized for preventing thrombosis, inflammation, and in-stent restenosis [21]. In another study, Italia *et al.* suggested the potential of biodegradable nanoparticles to improve the therapeutic efficacy of EGCG [22]. Singh *et al.* studied EGCG and the anticancer drug cisplatin as a combinatorial therapy in human cancer lines A549 (lung carcinoma), HeLa (cervical carcinoma), and THP-1 (acute monocytic leukemia). The results showed that the polyphenols alone or in combination with cisplatin were more effective in inhibiting cell proliferation, metastasis, angiogenesis, and apoptosis. Thus, it potentially could have a synergistic effect on other cancer drugs in treatment of various cancers. Results from another study showed the feasibility of using poly(lactide-co-glycolide)-PEG, (PLGA-PEG), nanoparticles encapsulating EGCG, functionalized with a small organic molecule (prostate-specific membrane antigen (PSMA) inhibitor) on the surface to enhance ECGC delivery specifically to prostate cancer cells. Through *in vitro* analysis, it was concluded that targeted delivery of EGCG via the PSMA-targeted nanoparticles was in general lower than the same nanoparticles without PSMA in LNCaP cells [14].

From the patient point of view and compliance, an oral route is the most convenient drug administration route. It is generally acknowledged that protection of the natural active ingredients against degradation in the harsh conditions of the gastrointestinal tract is one of the major obstacles for bioavailability of these products. Furthermore, the active ingredients of the natural products face two barriers that prevent them from reaching the human blood stream in large quantities: (i) physical barriers made up of the intestinal epithelium; (ii) enzymatic barriers of the intestinal tract that have the ability to degrade active ingredients. Therefore, it is of extreme importance to resolve the problem of oral consumption of nano-encapsulated EGCG and other natural products by incorporating biodegradable polymers suitable for oral consumption as the starting material, which will be more stable in the acidic environment of the gut and slowly release the active agent for absorption. We have observed a sustained release phenomenon for EGCG by optimizing its nano-encapsulated preparation for oral delivery (unpublished data).

## 3. Curcumin

Another natural product that has been extensively studied in nanotechnology applications is curcumin, the principal curcuminoid of the popular Indian spice turmeric (*Curcuma longa*), a plant typically grown and used in Southeast Asia [23]. It has been shown that curcumin can be used for the treatment of various cancers including multiple myeloma [24-26], pancreatic, myelodysplastic syndromes [27], colon [28,29], and psoriasis [27,30]. The potential anticancer activity of curcumin is explained by its capacity to exert apoptosis in cancer cells. However, major problems associated with its use are its low solubility, bioavailabity, and stability; curcumin is reported to be unstable in the gut [31], and trace amounts of curcumin that pass through the GI tract are rapidly degraded. Utilization of nanotechnology has proven to be very effective in solving this problem.

In a pioneering study by Bisht *et al.* [32], cross-linked and random copolymers of N-isopropylacrylamide (NIPAAM), with N-vinyl-2-pyrrolidone (VP) and poly(ethylene glycol) monoacrylate (PEG-A) nanoparticles, effectively incorporated curcumin. The size of these nanoparticles can be obtained within a range of 50 nm in diameter. The data demonstrated a comparable *in vitro* therapeutic efficacy of nanoformulated curcumin and free curcumin against a panel of human pancreatic cancer cell lines, as assessed by cell viability and clonogenicity assays. Further, nanoformulated curcumin's mechanism of action mirrored that of free curcumin, including induction of cellular apoptosis, blockade of nuclear factor kappa B (NFκB) activation, and downregulation of steady state levels of multiple pro-inflammatory cytokines (IL-6, IL-8, and TNFα). Most of the results demonstrated that both formulations of curcumin are equally potent with the nanoformulated curcumin, demonstrating some better results at lower doses [32]. In another study, Sahu *et al.* [33] synthesized a novel polymeric amphiphile with methoxy poly(ethylene glycol) (mPEG) as the hydrophilic segment, and palmitic acid (PA) as the hydrophobic segment. The conjugate, prepared in a single-step reaction, showed minimal toxicity on HeLa cells. This study only suggested a mechanism to make a hydrophobic drug like curcumin readily soluble in an aqueous system, but failed to demonstrate any dose advantage of curcumin in nanoformuation.

Thangapazham *et al.* [34] demonstrated a better efficacy of nanoformulated curcumin over free curcumin. They enhanced targeted delivery of curcumin for prostate cancer treatment by incorporating curcumin into liposomes coated with PSMA antibodies. The antiproliferative activity of liposomal curcumin was studied using two human prostate cancer cell lines, LNCaP and C4-2B. Treatment of cells with liposomal curcumin resulted in at least 70–80% inhibition of cellular proliferation without affecting their viability, with a 10-fold dose advantage over free curcumin [34]. In another study curcumin was nanoformulated with three biocompatible polymers (alginate, chitosan, and pluronic) by ionotropic pre-gelation followed by polycationic cross-linking. Pluronic F127 was used to enhance the solubility of curcumin in the alginate-chitosan nanoparticles. They demonstrated cellular internalization of curcumin-loaded composite nanoparticles [35]. It has also been demonstrated that curcumin-loaded poly(caprolactone) nanofiber matrix is bioactive, and has potential as a wound dressing with reduced inflammatory induction and an increased rate of wound closure [36]. PEGylated curcumin conjugate has been shown to have much more potent effects on pancreatic cancer cell growth inhibition than free curcumin [37].

The potential of using nanoparticles for oral delivery of curcumin has been demonstrated by preparing nanoformulated curcumin by an emulsion technique. The results suggest that the *in vitro* release of curcumin was predominantly by a diffusion phenomenon and followed Higuchi's release pattern. The pharmacokinetics revealed that curcumin-entrapped nanoparticles demonstrate at least a 9-fold increase in oral bioavailability when compared to curcumin administered with piperine as an absorption enhancer [38]. Thus, in this study, a composite nanoparticle was prepared by using three biocompatible polymers, alginate (ALG), chitosan (CS), and pluronic by ionotropic pre-gelation followed by polycationic cross-linking nanoformulation of curcumin with a tripolymeric composite for delivery to cancer cells. Encapsulation efficiency of curcumin in composite nanoparticles showed a considerable increase over ALG-CS nanoparticles without pluronic. These composite nanoparticles were observed to have a suitable size distribution, drug encapsulation efficiency, and drug release kinetics. The half-maximal inhibitory concentrations for free curcumin and encapsulated curcumin were found to be 13.28 μM and 14.34 μM, respectively [39].

Another important study in the arena of nanoformulated curcumin came from Aggarwal *et al.* They encapsulated curcumin with over 97.5% efficiency in PLGA and PEG [40]. They observed that *in vitro*, nanoformulated curcumin exhibited very rapid and more efficient cellular uptake than free curcumin. Nanoformulated curcumin was also seen to be at least as potent as (and possibly more potent than) curcumin in inducing apoptosis of leukemic cells and in suppressing proliferation of various tumor cell lines. When examined by electrophoretic gel shift mobility assay, curcumin nanoparticles were more active than curcumin in inhibiting TNF-induced NF-κB activation and in suppression of NF-κB-regulated proteins involved in cell proliferation (cyclin D1), invasion (MMP-9), and angiogenesis (VEGF). In mice, curcumin nanoparticles were more bioavailable and had a longer half-life than curcumin. Mukherjee and Vishwanathan showed the successful formation of smooth and spherical curcumin-loaded PLGA nanospheres with an encapsulation efficiency of around 91%. The results demonstrated a robust intracellular uptake of the nanospheres in prostate cancer cells, and the cell viability studies revealed that the curcumin-loaded nanospheres were able to exert a more pronounced effect on the cancer cells as compared to free curcumin [41].

More recently, efficacious formulations of curcumin, including nanocrystal solid dispersion (CSD-Cur), amorphous solid dispersion (ASD-Cur), and nanoemulsion (NE-Cur), were designed with the aim of improving physicochemical and pharmacokinetic properties. All curcumin formulations exhibited marked improvement in the dissolution behavior when compared with crystalline curcumin. Significant improvement in pharmacokinetic behavior was observed in the newly developed formulations, as evidenced by a 12-fold increase (ASD-Cur), 16-fold increase (CSD-Cur), and a 9-fold increase (NE-Cur) of oral bioavailability [42]. In a study that investigated the self-organized, mixed assemblies of curcumin, curcumin was assembled together with a poly(oxyethylene) cholesteryl ether (PEG-Chol) to form nano-sized assemblies (around 10 nm) of assumed micelles. In contrast with the rapid decomposition of free curcumin due to hydrolysis, the curcumin was highly stabilized in the nanoparticles, especially below 40 mol% curcumin. A cell viability assay revealed that the cytotoxic activity of the curcumin/PEG-Chol nanoparticles against myeloma cells is higher than that of free curcumin in a comparison at 1 μM. On the other hand, both the curcumin/PEG-Chol nanoparticles and PEG-Chol micelles had significant cytotoxicity to the myeloma cells at 5 μM [43].

In a study undertaken to enhance the bioavailability of curcumin along with reducing the required dose through selective targeting to colon, the polymer Eudragit S100 was used to aid in targeting since it dissolves at colonic pH to result in selective colonic release of the entrapped drug. Nanometric, homogeneous, spherical particles were synthesized with an encapsulation efficiency of around 72%. The cell growth inhibition in the HT-29 cell line was almost double by nanoparticles as compared to curcumin alone at all tested concentrations [44]. More recently two studies by Yallapu *et al.* [45,46] tested the efficacy of nanoformulated curcumin for cancer therapeutics. In the first study [45] curcumin was encapsulated in PLGA nanoparticles in the presence of poly(vinyl alcohol) and poly(L-lysine) stabilizers. It was observed that the optimized curcumin nanoformulation, compared to free curcumin, had a 2-fold increase in the cellular uptake performed in cisplatin-resistant A2780CP ovarian cells and a 6-fold increase in the cellular uptake performed in cisplatin-resistant metastatic MDA-MB-231 breast cancer cells. This effect was correlated with enhanced apoptosis induced by the nanoformulated curcumin. Further, the antibody conjugation compatibility of our PLGA-nanoparticle formulation was also demonstrated [45]. In the second study, a PLGA nanoformulation of curcumin was successfully generated, and steady and prolonged release of curcumin, antibody conjugation capability, and effective inhibition of ovarian cancer cell growth was successfully tested [46].

Gupta *et al.* [47] reported a synthesis of nanoparticles encapsulating curcumin, blending biopolymers like silk fibroin and chitosan. The efficacy of these nanoparticles was tested *in vitro* in breast cancer cell lines. It was observed that silk fibrin-coated nanoparticles were superior in entrapment efficiency, and showed better cellular uptake than the silk-fibrin-chitosan blended nanoparticles. Also, the silk-fibrin blended nanoparticles reduced the viability of Her2/*neu* high-expressing breast cancer cells. It was hypothesized that curcumin has the ability to suppress Her2 and NF-κB pathways in breast cancer cells

In a novel approach, the utilization of PLGA nanoparticles for incorporation of multiple drugs was demonstrated recently [48]. Curcumin and doxorubicin were coencapsulated in PLGA nanoparticles and the synergistic effect was examined in multi-drug resistant K562 leukemia cells. The dual drug-loaded nanoparticles were effectively delivered into K562 cells, and the combination of the drugs was capable of inducing apoptosis even if in a lower concentration compared to either drug alone in both solution or in formulation. It was also shown that this formulation has the ability to increase the cytotoxcity of the drug, enhancing apoptosis and thus shows the synergistic effect. There is other evidence of the use of nanoparticles incorporating curcumin for treatment of different types of cancer cell lines like glioma (C6) [49], human colon tumor cells, human pancreatic carcinoma cells [50], and hepatocellular carcinoma cells [51]. In most cases this water insoluble product (curcumin) was successfully incorporated in the nanformulation and has the potential to be used as an injectable formulation in the near future. However, most of the studies were limited to the *in vitro* or small animal studies to determine the anticancer efficacy as a proof of concept. These studies suggested that curcumin could be successfully formulated by utilizing nanotechnology, and the resulting formulations were demonstrated to have better efficacy by showing their potential against a variety of cancer cells, and further suggested better bioavailability under *in vivo* situations. However, more studies are certainly required to take the research into clinical practice.

## 4. Resveratrol

Resveratrol (3,5,4′-trihydroxy-*trans*-stilbene) is a naturally occurring phenol that can act as a phytoalexin, used by several plants to help fight and then repair attacks by external photogens like bacteria and fungi. Resveratrol is also a well-known antioxidant. Resveratrol can work through many of the intracellular signaling pathways, including cell survival and apoptosis modulator tumor angiogenesis and metastatic switches [52]. Thus, it has tremendous potential to be used as an anticancer drug. The anticancer activity of resveratrol was first demonstrated by Jang *et al.* in 1996 [53] for the prevention of skin cancer development in mice. Since then there has been tremendous effort to use resveratrol as an anticancer agent in various cancer models, including skin cancer [54-56], breast cancer [57-59], fibrosarcoma [60,61], lung cancer [62,63], gastric and colorectal cancer [64], prostate cancer [65,66], hepatoma [67,68], neuroblastoma [69], pancreatic cancer [70,71], and leukemia [72,73]. However, most of these studies' results have failed to be replicated in humans, mainly due to resveratrol's very short half-life. It is rapidly glucoronated and sulfonated, and is a lipophilic agent, and thus it has failed when tested in the clinic.

Nanotechnology-based approaches are currently being tried to enhance the bioavailability of resveratrol; significant progress has been made. The first nanoformulation of resveratrol was performed with chitosan nanoparticles. That study suggested that chitosan nanoformulations have a sustained release *in vitro*. The rate of release was slowed down with the increase of solidification agents [74]. In another study, resveratrol-loaded nanoparticles at lower concentration led to significantly higher cell death as compared to an equivalent dose of free resveratrol, and this difference of cytotoxicity was not found to be abrogated by the inclusion of Vitamin E [75]. Another study's results suggested that 12 hour pre-incubation of resveratrol-loaded nanoparticles protects cells from beta-amyloid peptide (Abeta)-induced damage in a dose-dependent manner by attenuating intracellular oxidative stress and caspase-3 activity [76].

Shao *et al.* [75] showed the feasibility of incorporating resveratrol in mPEG-poly(epsilon-caprolactone)-based nanoparticles with high entrapment efficiency. They found that even a low concentration of encapsulated resveratrol can lead to significantly higher cell death compared to an equivalent dose of free resveratrol. It was speculated that the differential cytotoxicity between resveratrol and resvertrol-loaded nanoparticles may be mediated by the discrepancy of intracellular reactive oxygen species (ROS) levels. The results suggest that resveratrol-loaded nanoparticles could be a potentially useful chemotherapeutic formulation for malignant glioma therapy, and may have clinical relevance in the near future.

Nanosuspensions of resveratrol (5%) were produced for a dermal application; four nanosuspensions were investigated using the stabilizers Tween 80, Poloxamer 188, Plantacare 2000, and Inutec SP1. Nanocrystal sizes were about 150 nm (Poloxamer, Plantacare), and about 200 nm (Tween, Inutec), and no amorphous fraction was detected. In a short-term stability study (30 days, room temperature), the nanosuspensions with 2% w/v with the above-mentioned four stabilizers proved to be either less stable or at least had no stability advantage over the 1% formulations (with all the above-mentioned stabilizers). Formulations with 1% stabilizer were stable in the short-term study, and Plantacare and Inutec demonstrated the best stabilization [77].

Solid lipid nanoparticles (SLN) have been used as a carrier for resveratrol (RSV) [52]. The effects of SLN, empty or loaded with resveratrol (SLN-RSV), on the internalization, growth, morphology, metabolic activity, and genetic material of keratinocytes were compared to those of resveratrol in solution. Fluorescence images clearly showed that SLN with a size below 180 nm move promptly through the cell membrane, distribute throughout the cytosol, move successively among different cellular levels, and localize in the perinuclear region without inducing any cytotoxicity. Resveratrol solubility, stability, and intracellular delivery were all increased by loading into SLN. The release profile of resveratrol showed a biphasic pattern, reflecting its distribution in SLN. Resveratrol in solution was slightly cytotoxic. That was prevented by loading it into SLN, which preserved cell morphology. The cytostatic effect of SLN-RSV was much more expressed than that of resveratrol in solution. Delivery of resveratrol by SLN contributes to effectiveness of resveratrol on decreasing cell proliferation, with potential benefits for prevention of skin cancer [52].

The antitumor effects of resveratrol in a nanoformulation with bovine serum albumin (BSA) nanoparticles have been investigated [78]. The administration of resveratrol-BSA nanoparticles significantly inhibited the growth rate of subcutaneously implanted human primary ovarian cancer cells SKOV(3) after three weeks in nude mice compared to free resveratrol. Although not much progress has been made yet on the nanotechnology-based anticancer potential of resveratrol, it should be noted that this field is still very primitive, and thus more research is required to be able to achieve real benefits from this anticancer agent.

Narayanan *et al.* [79] used liposome-encapsulated curcumin and resveratrol individually and in combination in male B6C3F1/J and prostate-specific PTEN knockout mice. *In vitro* assays using PTEN-CaP8 cancer cells were also done to investigate the combined effects of curcumin with resveratrol. HPLC analysis of serum and prostate tissues showed a significant increase in curcumin level when liposome-encapsulated curcumin was co-administered with liposomal resveratrol. Combination of liposomal forms of curcumin and resveratrol significantly decreased prostatic adenocarcinoma *in vivo* in PTEN mice and the *in vitro* studies revealed that curcumin plus resveratrol effectively inhibited cell growth and induced apoptosis. Findings from this study for the first time provided evidence on natural products in combination to enhance chemopreventive efficacy in prostate cancer.

## 5. Taxol

Taxol, a potent anticancer agent, has stimulated an intense research effort in recent years. In humans it has been shown to have activity against a number of leukemias and solid tumors in the breast, ovary, brain, and lung [80-83]. Taxol was isolated from the bark of the Pacific Yew tree in 1971, and is among the first FDA-approved chemotherapy drugs that originated from natural sources. Its brand name is Paclitaxel, coined when it was developed commercially by Bristol-Myers Squibb; it is sold under the trademark name Taxol.

Despite its clinical and commercial success, Taxol suffers from the fact that it is water insoluble. Ethanol or Cremophor EL are generally used for solubilization, but they have their own side effects. A study was done to develop a polymeric drug delivery system for Paclitaxel, intended to be intravenously administered, capable of improving its therapeutic index, and devoid of the adverse effects of Cremophor EL [84]. Paclitaxel-loaded PLGA nanoparticles were prepared by the interfacial deposition method. The release behavior of Paclitaxel from the developed nanoparticles exhibited a biphasic pattern characterized by an initial, fast release during the first 24 hours, followed by a slower, continuous release. The *in vitro* results demonstrated that incorporation of Paclitaxel in nanoparticles strongly enhances the cytotoxic effect of the drug as compared to Taxol, this effect being more relevant for prolonged incubation times [84].

Feng *et al.* formulated Paclitaxel in PLGA nanoparticles by a modified solvent extraction/evaporation technique [85]. It was found that these natural emulsifiers have great advantages for nanoparticle formulation of Paclitaxel over the traditional macromolecular emulsifiers, such as polyvinyl alcohol. The results also suggested that the formulation could be modified to achieve drug encapsulation efficiency as high as 100%, and the release kinetics can be made under control. The HT-29 cancer cell line experiment showed that after 24 hours of incubation, cell mortality caused by Paclitaxel administered by such a nanoparticle formulation could be more than 13 times higher than that caused by free Paclitaxel under similar conditions [85].

Another study [86] determined the efficacy of Paclitaxel loaded in sterically-stabilized, biocompatible, and biodegradable phospholipid nanomicelles (SSMM) and actively-targeted, vasoactive, intestinal, peptide-grafted SSMM (SSMM-VIP) in circumventing P-glycoprotein (P-gp)-mediated Paclitaxel resistance in BC19/3 cells, a human breast cancer cell line that expresses >10-fold higher P-gp than its parental sensitive cell line, MCF-7. The study found that in drug-sensitive MCF-7 cells, Paclitaxel-loaded SSMM (P-SSMM), and Paclitaxel-loaded SSMM-VIP (P-SSMM-VIP) significantly inhibited cell growth in a dose-dependent fashion (p < 0.05). Both formulations were approximately 7-fold more potent than Paclitaxel dissolved in DMSO (P-DMSO) with P-SSMM and P-SSMM-VIP showing similar efficacy. By contrast, in drug-resistant BC19/3 cells, P-SSMM-VIP was significantly more effective than either P-SSMM or P-DMSO (approximately 2-fold and 5-fold, respectively; p < 0.05) [86].

In an effort to determine efficacy, nanoparticulate Paclitaxel (NPs-Tx) was evaluated for antiproliferative activity in a human prostate cancer cell line (PC-3), and for its effect on tumor inhibition in a murine model of prostate cancer. It was observed that, under *in vitro* conditions, the nanoparticles exhibited sustained release of the encapsulated Paclitaxel (60% release in 60 days). The IC_50_ of the drug with transferrin (Tf)-conjugated nanoparticles was about 5-fold lower than with unconjugated nanoparticles or drug in solution. Animals studies, in which mice received a single-dose intratumoral injection of NPs-Tx-Tf (Tx dose = 4 mg/kg) demonstrated complete tumor regression and greater survival rate than those mice that received either NPs-Tx or Paclitaxel-Cremophor EL formulation (conventionally used Paclitaxel formulation) [87].

A novel, highly water-soluble poly(L-γ-glutamyl-glutamine)-Paclitaxel nanoconjugate (PGG-PTX) has been developed. The potency of PGG-PTX when tested *in vitro* against the human lung cancer H460 cell line was comparable to other known polymer-PTX conjugates. However, PGG-PTX possessed lower toxicity compared with PGGPTX in mice. The maximum tolerated dose of PGG-PTX was found to be 350 mg PTX/kg, which is 2.2-fold higher than the maximum tolerated dose of 160 mg PTX/kg reported for the PGA-PTX [88]. In a different study, cationic micellar nanoparticles self-assembled from a biodegradable amphiphilic copolymer were used to deliver human TRAIL (Tumor Necrosis Factor-Related Apoptosis-Inducing Ligand) and Paclitaxel simultaneously [89]. Polyplexes formed between Paclitaxel-loaded nanoparticles and TRAIL were observed to be stable, with a size of about 180 nm and a zeta potential at about 75 mV. Anticancer effects and apoptotic pathway mechanisms of this drug-and-protein co-delivery system were investigated in various human breast cancer cell lines with different TRAIL sensitivity. The co-delivery nanoparticulate system induced synergistic anticancer activities with limited toxicity in noncancerous cells [89].

## 6. Camptothecin

Camptothecin (CPT), a cytotoxic alkaloid isolated from *Camptotheca acuminate*, was discovered in 1966 by Wall and Wani [90]. It has been observed that CPT and its derivatives inhibit religation of cleaved, single-stranded DNA by targeting the nuclear enzyme topoisomerase I (Top I). This finally leads to inhibition of replication, and cell death occurs. Though CPT and CPT derivatives exhibit excellent anticancer activity, the major problem associated with this kind of drug is its extreme lypophilicity and instability of the lactone ring. The most prominent drugs approved by the FDA in this category of water soluble derivatives of CPT are Camptosar^®^ (irinotecan hydrochloride, or CPT-11) and Hycamtin^®^ (topotecan), and they can be used for advanced colorectal carcinoma and ovarian cancers, respectively. There have been many studies involving nanoparticle-mediated delivery of CPT and its analog to make injectable formulations to treat different cancers [91].

Onishi *et al.* [92] used PLGA nanoparticles to incorporate irinotecan. Nanoparticles containing irinotecan of size in-between 80 to 210 nm inhibited tumor growth considerably in a mouse model bearing subcutaneous Sarcoma 180 with a repeated dose of 20 mg/kg. It was also demonstrated that this nanoformulation had the ability to maintain a higher concentration of the drug for a longer residence time, accounting for the better suppression of the tumor. Since that study, PLGA nanoparticles have been one of the major carriers used to deliver CPT and its analogs. In 2007, it was demonstrated that PLGA [93] nanoparticles can be used as an efficient delivery vehicle for 9-nitrocamptothecin (9-NC) in an attempt to develop a formulation that can be administered intravenously to increase the therapeutic index. Though *in vitro* drug release kinetics and other physical characterizations were performed, the efficacy of the drugs is unknown.

The *in vitro* targeted capability of PLGA nanoparticles loaded with camptothecin and comprised of a layer of peripheral antibodies against colorectal tumor cells was demonstrated by MacCarron *et al.* [94]. Observation of fluorescently labeled nanoparticles confirmed the increased uptake of the antibody-nanoparticles compared to the nanoparticles without the antibody. It was also observed that targeted delivery of the nanoparticles by the antibody in the HCT116 cell line increased the cytotoxcity of camptothecin. Another study used PLGA-PEG-folate (PLGA-PEG-FOL) nanoparticles for targeted delivery of a camptocatchein analog, SN-38, (7-ethyl-10-hydroxycamptothecin), an active metabolite of irinotecan, via folate receptor-mediated targeted delivery [95]. It was observed that targeted delivery of SN-38 via PLGA-PEG-FOL nanoparticles had significantly higher cytotoxcity than the nanoparticles without the folic acid component. In an interesting study by Williams *et al.* [96], it was observed that the efficacy of SN-38 is dependent on the size of the lipid-based nanoparticles used as a delivery vehicle. That *in vivo* study in nude mice bearing colon adenocarcinoma xenografts (HT-29) revealed that better tumor suppression and longer survival rates (65 days) were achieved by using nanoparticles with a size around 375 nm in diameter containing SN-38, compared to using nanoparticles with a size of 100 nm containing SN-38 (48 days), and free SN-38 (51 days). The half-life of the SN-38 also increased when it was administered in the nanoformulation compared to the free drug.

Min *et al.* [97] synthesized hydrophobically modified glycol chitosan (HGC) encapsulating a water-insoluble CPT. These HGC-CPT nanoparticles had a size around 280 to 330 nm in diameter, and had a significantly higher loading efficiency (80%). The anticancer efficacy of the HGC-CPT nanoparticles was evaluated in mice bearing subcutaneously implanted MDA-MB231 xenograft. The intraveneous injection of the nanoformulation seemed to suppress tumor growth when it was compared to the free dose. *In vivo* monitoring of the dye-labeled nanoparticles confirmed that there was higher accumulation of the nanoparticles at the tumor site. It was hypothesized that the increase of the therapeutic effect of CPT when incorporated in nanoparticles might be because of the higher accumulation of CPT at the tumor site.

An attempt was made to evaluate the *in vivo* efficacy of CPT when incorporated in cyclodextrins or polymeric nanoparticles like PLGA and poly-ε-caprolactone (PCL) [98]. The efficacy of the nanofomulation was evaluated in malignant glioma (9L) tumor cells. Among all different types of nanoparticles synthesized by nanoprecipitation methods, it was observed that 6-O-Capro-β-CD nanoparticles had the highest entrapment efficiency as well as a better controlled-release profile. Additionally, *in vitro* and *in vivo* studies showed that 6-O-capro-β-CD cyclodextrin and concentrated 6-O-capro-β-CD cyclodextrin nanoparticles drastically suppressed the growth of 9L glioma tumors and increased the survival rate.

There have been tremendous efforts made to use nanoparticles to increase the solubility and bioavailability of these poorly water soluble natural products and their analogs. However, these efforts are in a very nascent stage and most of the experiments are limited to the laboratory. Proper use of biodegradable, safe polymers or nanomaterials to incorporate CPT and CPT analogs might bring this cutting edge technology into practice in treating cancer patients in the future.

## 7. Other Natural Products

There has been limited investigation of the use of pomegranate extract or other natural ingredients in the treatment and prevention of various cancers, but there is great potential that some of the components of pomegranate can be utilized for effective cancer prevention and treatment. There have been a few reports that that pomegranate extracts selectively inhibit the growth of breast [99,100], prostate [101-103], colon [104], lung tumors, and skin cancer both in culture plates and mice xenograft model. There have been few reports of phase II clinical trials of pomegranate juice in patients with prostate cancer to increase the lifetime of the patient. However, a major problem associated with pomegranate extract and its active ingredient is its instability and poor bioavailability. Nanotechnology has the potential to solve this problem by masking the active ingredient inside the nanoparticulate network and thus preventing further degradation, and increasing the bioavailability. In a recent study by Li *et al.* [105], gelatin nanoparticles incorporating partially purified pomegranate (PPE) ellagitannins were synthesized. The loading efficiency of the various ingredients like punicalagin A and punicalagin in the extract was measured after incorporation into genmatin nanoparticles, and it was found that many of the active ingredients of the extract were incorporated in the nanoparticles with entrapment efficiency. However, the PPE-gelatin nanoparticle suspension was less effective than PPE alone in inducing the early stage of apoptosis on human promyelocytic leukemia cells (HL-60). But they had similar effects in inducing a late stage of apoptosis and necrosis. In our laboratory we have been actively involved in synthesizing biodegradable nanoparticles incorporating pomegranate extract and green tea extract for treatment of various cancers, including breast cancer and hapatocelluar carcinoma, and preliminary results are very promising. In a recent study by Narayanan *et al.* [106] incorporating grape seed extract (GSE) in PLGA nanoparticles, folic acid was conjugated to the nanoparticles for targeted delivery to the tumor cell line overexpressed folate receptor. The IC(50) of the GSE was lowered approximately 3 times compared to the free counterpart, indicating the specificity of the nanocarrier for cancer cells.

## 8. Conclusions and Future Prospects

Proponents of nanotechnology consider this technology as one of most powerful tools in modern society; it has made a revolutionary impact on every aspect of human life. Looking at the speed of advancement of nanomedicine, particularly utilization of various nanoparticles in prevention, diagnosis, and treatment of a complex disease like cancer, one can predict that this cutting edge technology will be on our doorstep soon.

Our proof-of-principle study [12] demonstrated the usefulness of nanoparticulate technology to enhance the therapeutic effectiveness of natural agents, EGCG in our case. Based on our study, the concept was utilized by researchers worldwide and as described herein the outcomes of the studies are very convincing. Nanotechnology-mediated delivery of natural products is effective because nanoparticles rarely pose any toxicity to normal cells [107]. Moreover, being biodegradable, these nanoparticles are considered to be safe [15]. Ours and others' studies suggest that nanotechnology could be utilized with considerable advantage over currently employed chemopreventive and chemotherapeutic approaches for cancer. Apart from the nanochemoprevention side of nanotechnology, studies have shown that nanotechnology is a plausible approach for diagnosis, imaging, and therapeutics. Considerable research is now being devoted to nanoparticles-based drug delivery. Several nanotechnology-based constructs are currently in clinical or preclinical development and several of these are already approved by the FDA (Table 1).

Advancements in utilization of nanotechnology might help us to achieve the higher concentrations of the natural products necessary for efficacy against various diseases. It is assumed that a cure for cancer will be available by the year 2015 [108,109], and it is also anticipated that nanotechnology will be a $1 trillion industry by that time, with most of the impact focused on healthcare and cancer therapy. Though there has been concern about the general safety and environmental effects, and potential health effects on those involved in manufacturing nanotechnologies, these issues can be addressed in the course of time. Abraxane® is one of the major success stories of a nanomedicine approach to treat cancer. As an injectable suspension, Abraxane® evades the hypersensitivity reaction associated with Cremophor EL, the solvent in traditional Paclitaxel, and thus has been successful in addressing the solubility problem associated with Paclitaxel. In conclusion, we can say that nanotechnology combined with multifunctional nanocarriers with tumor-specific ability carrying one or multiple natural products has the potential to be within reach to treat cancer in near future.

## Figures and Tables

**Table 1.** Examples of nanocarrier-based drugs on the market.

**Product**	**Company**	**Drug**	**Formulation**	**Application**
Doxil	Sequus Pharmaceuticals	doxorubicin	Pegyalated liposome	Metastatic ovarian cancer and Kaposi sarcoma in AIDS patients
Dauno-Xome	NeXstar Pharmaceutica	daunorubicin citrate	liposome	Kaposi sarcoma in AIDS patients
Emend	Merck/Elan	aprepitant (MK 869)	proprietary Nanocrystal® formulation	Chemotherapy-related side effects (nausea and vomiting)
Abraxane	Abraxis Bioscience AstraZeneca	Paclitaxel	albumin nanoparticles	Metastatic breast cancer
Myocet	Zeneus Pharma Sopherion Therapeutics	doxorubicin	liposome	Combinatorial therapy of breast cancer, ovarian cancer, and Kaposi sarcoma
Combidex	Advanced Magnetic Inc.	iron oxide	Dextran10 coated iron oxide nanoparticles	Different tumor imaging
Oncaspar	Enzon	L-asparaginase	Polymer protein conjugate	Leukemia
